# Reconstitution of a mini‐gene cluster combined with ribosome engineering led to effective enhancement of salinomycin production in *Streptomyces albus*

**DOI:** 10.1111/1751-7915.13686

**Published:** 2020-12-03

**Authors:** Dong Li, Yuqing Tian, Xiang Liu, Wenxi Wang, Yue Li, Huarong Tan, Jihui Zhang

**Affiliations:** ^1^ State Key Laboratory of Microbial Resources Institute of Microbiology Chinese Academy of Sciences Beijing 100101 China; ^2^ College of Life Sciences University of Chinese Academy of Sciences Beijing 100049 China

## Abstract

Salinomycin, an FDA‐approved polyketide drug, was recently identified as a promising anti‐tumour and anti‐viral lead compound. It is produced by *Streptomyces albus*, and the biosynthetic gene cluster (*sal*) spans over 100 kb. The genetic manipulation of large polyketide gene clusters is challenging, and approaches delivering reliable efficiency and accuracy are desired. Herein, a delicate strategy to enhance salinomycin production was devised and evaluated. We reconstructed a minimized *sal* gene cluster (mini‐cluster) on pSET152 including key genes responsible for tailoring modification, antibiotic resistance, positive regulation and precursor supply. These genes were overexpressed under the control of constitutive promoter P_kasO*_ or P*_neo_*. The *pks* operon was not included in the mini‐cluster, but it was upregulated by SalJ activation. After the plasmid pSET152::mini‐cluster was introduced into the wild‐type strain and a chassis host strain obtained by ribosome engineering, salinomycin production was increased to 2.3‐fold and 5.1‐fold compared with that of the wild‐type strain respectively. Intriguingly, mini‐cluster introduction resulted in much higher production than overexpression of the whole *sal* gene cluster. The findings demonstrated that reconstitution of *sal* mini‐cluster combined with ribosome engineering is an efficient novel approach and may be extended to other large polyketide biosynthesis.

## Graphical abstract

A novel *sal* mini‐gene cluster was designed to improve salinomycin production in *S. albus*. *pks* operon omitted from mini‐cluster was up‐regulated by activator SalJ. Mini‐cluster combined with ribosome engineering boosted salinomycin production.

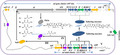

## Introduction

Clinical use of penicillin and streptomycin has revolutionized the treatment of infectious diseases. Numerous antibiotics were thenceforth discovered from the natural environmental bioresource. Among them, *Streptomyces* became the major source of commercially important antibacterial agents due to the tremendous varieties of their secondary metabolites (Barka *et al*., 2016; Liu *et al*., 2018). Because of improper use and abuse of antibiotics as well as horizontal transfer of antibiotic resistance genes between bacteria by conjugation, transduction or transformation, these have led to the appearance of antibiotic resistance and the loss of antibiotic native efficiency. Moreover, with the expansion of clinical application, antibiotics supply becomes imperative for health care, but high‐yield producing strains are still urgently needed in both academic and industrial fields.

Rational design and refactoring of biosynthetic pathways with synthetic biology and metabolic engineering strategies proved to be effective for strain optimization (Liu *et al*., 2013; Palazzotto *et al*., 2019). However, single gene manipulation sometimes is insufficient to boost the production and often leads to accumulation of intermediates. In contrast, strategies directing to all the essential genes involved in key biosynthetic pathways have been exploited based on the technological advances in cloning and editing large DNA fragments, such as TAR (transformation‐associated recombination), LLHR (linear–linear homologous recombination), CATCH (Cas9‐assisted targeting of chromosome segments) and ϕBT1 *attP*‐*attB*‐*int* system (Li *et al*., 2017; Zhuo *et al*., 2017; Zheng *et al*, 2019). The common issue in cloning and manipulation of gene clusters is that the efficiency would substantially decrease with the increase of target DNA fragment size apart from certain inherent limitations when the technique is employed. Another challenge in engineering strains is to balance the expression of individual biosynthetic gene modules to relieve the bottleneck steps of the pathway, while it is hard to overcome by either single gene manipulation or complete gene cluster expression, especially for the large gene clusters.

Polyketides are a major family of antibiotics with special biosynthetic pathways, and the responsible gene clusters over 100 kb in size widely exist in *Streptomyces* genomes, such as the cluster of monensin (97 kb), rapamycin (107.3 kb), amphotericin (113.2 kb) or nystatin (123.6 kb) (Schwecke *et al*., 1995; Brautaset *et al*., 2000; Caffrey *et al*., 2001; Oliynyk *et al*., 2003). Many polyketide compounds have been developed as important anti‐pathogen, anti‐tumour or immunosuppressive agents used broadly in the fields of pharmaceuticals and agrochemicals. Exploring more efficient and universal genetic manipulation system suitable for this kind of gene clusters is highly expected. As a pioneering work in conceptual research, salinomycin produced by *Streptomyces albus*, a polyketide antibiotic and a promising drug candidate targeting cancer stem cells (Gupta *et al*., 2009) and SARS‐CoV‐2 (Ianevski *et al*., 2020; Pindiprolu *et al*., 2020), was chosen to establish an alternative method for improving the yield. Salinomycin gene cluster (*sal*) spanning over 104 kb has been sequenced in *S. albus* DSM 41398 (GeneBank HE586118.1), and its biosynthetic pathway was proposed (Jiang *et al*., 2012; Yurkovich *et al*., 2012; Jiang *et al*., 2015; Luhavaya *et al*., 2015). The *sal* cluster contains at least 27 genes, including nine type I *pks* genes (*salAI* to *salAIX*, ˜ 78.6 kb) as a single transcriptional unit responsible for the biosynthesis of the polyketide scaffold, seven tailoring genes responsible for the modification of polyketide scaffold, and resistance and regulatory genes. Usually, regulators act as a switch to regulate the onset of antibiotic biosynthesis (Liu *et al*., 2013; Guan *et al*., 2019; Li *et al*., 2019a). So strategies towards regulatory system are of importance for those complex biosynthetic pathways. In terms of metabolic modulation‐related approaches, diligent efforts have been made to improve the production of antibiotics via various techniques (Lu *et al*., 2016; Tanaka *et al*., 2017; Zhang *et al*., 2017a, 2017b, 2019,2017a, 2017b, 2019). Also, the whole *sal* gene cluster was cloned and heterologously expressed in *S. coelicolor* A3(2) (Yin *et al*., 2015). However, there is no any report hitherto on the yield improvement via amplification of the whole *sal* cluster, and harnessing such a large gene cluster to achieve significant overexpression would be challenging due to the involvement of *pks* genes and multiple tailoring steps.

In this study, we describe a novel 'mini‐*sal* gene cluster (hereafter abbreviated as mini‐cluster)' strategy for efficiently improving salinomycin production. A constructed gene cluster contains important gene modules encoding key enzymes in the pathway to gain possible equivalent productivity as overexpressing the whole gene cluster. Also, the combinatory effect of mini‐cluster and ribosome engineering on salinomycin production was evaluated. This would provide an efficient novel approach for enhancing the expression of large polyketide gene clusters.

## Results and discussion

### Selection of compatible promoters for high‐yield salinomycin production

**Fig. 1:**
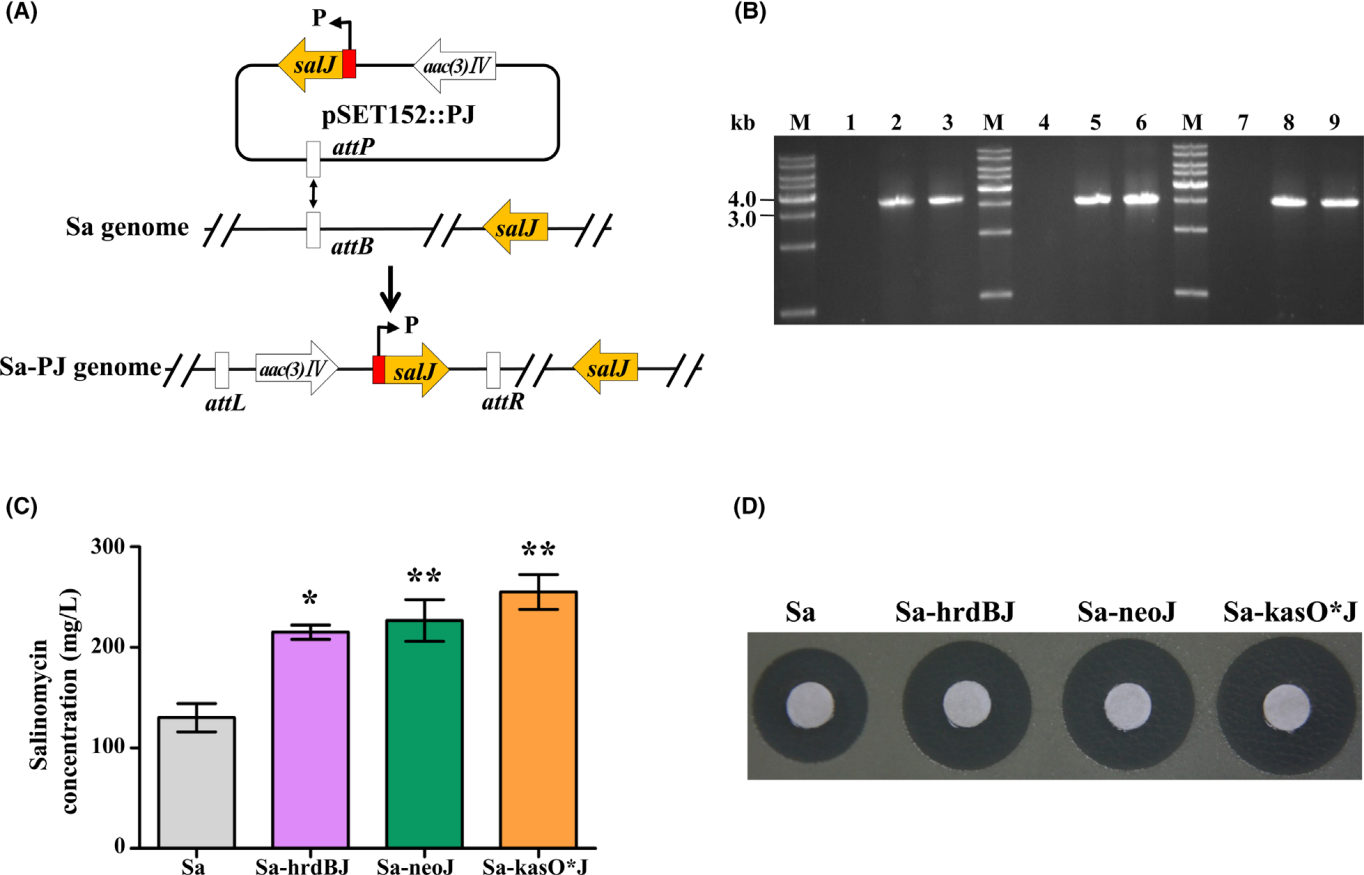
Effect of *salJ* overexpression under the control of different promoters on salinomycin production. A. Construction of *salJ* overexpression strains. P, promoter. Sa‐PJ indicates *salJ* overexpression strains under the control of different promoters. B. Genomic analysis of *salJ* overexpression strains by PCR. M, DNA ladder; lanes 1, 4 and 7: PCR amplification products using the genomic DNA of Sa as template; lanes 2‐3, 5‐6 and 8‐9: PCR products using genomic DNA of strains Sa‐hrdBJ (primer pair P*_hrdB_*‐F/salJ‐R), Sa‐neoJ (primer pair P*_neo_*‐F/salJ‐R) or Sa‐kasO*J (primer pair P*_kasO_* *‐F/salJ‐R) as template, and the sizes of the corresponding PCR products are 3333 bp, 3072 bp and 3002 bp respectively. C. HPLC analysis of salinomycin production from the fermentation broth of different strains. Data are presented as the averages of three independent experiments. Error bars indicate standard deviations (SD). Significant difference between the recombinant strains and wild‐type strain (Sa) was confirmed by Student’s *t*‐test (* and ** represent *P* < 0.05 and *P* < 0.01 respectively). D. Bioassays of salinomycin from the fermentation broth of different strains against *B. cereus*. Sa, *Streptomyces albus* CGMCC 4.5716 (wild‐type strain); Sa‐hrdBJ, Sa‐neoJ and Sa‐kasO*J are *salJ* overexpression strains.

To enhance salinomycin production, one of the conventional approaches is to increase the expression of key genes under control of suitable strong promoters, but the compatibility of different promoters with the strains needs to be evaluated first. *salJ* is a positive regulatory gene of salinomycin biosynthetic gene cluster (Zhu *et al*., 2016). To assess the activity of different promoters in *S. albus* CGMCC 4.5716, recombinant strains Sa‐hrdBJ, Sa‐neoJ and Sa‐kasO*J for overexpressing *salJ* under the control of strong promoters P*_hrdB_*, P*_neo_* (Du *et al*., 2013) and P*_kasO_* * (Wang *et al*., 2013) were constructed (Fig. 1A and B) respectively. In comparison with *S. albus* CGMCC 4.5716, salinomycin production increased 1.95‐fold in Sa‐kasO*J, 1.73‐fold in Sa‐neoJ and 1.65‐fold in Sa‐hrdBJ as determined by HPLC analysis (Fig. 1C), which was further verified by the bioassays against *B. cereus* CGMCC 1.1626 (Fig. 1D). The results indicated that P_kasO*_ and P*_neo_* are more compatible with *S. albus* CGMCC 4.5716, so they were selected for the subsequent strain construction.

### Effect of tailoring and resistance gene modules on salinomycin production

**Fig. 2:**
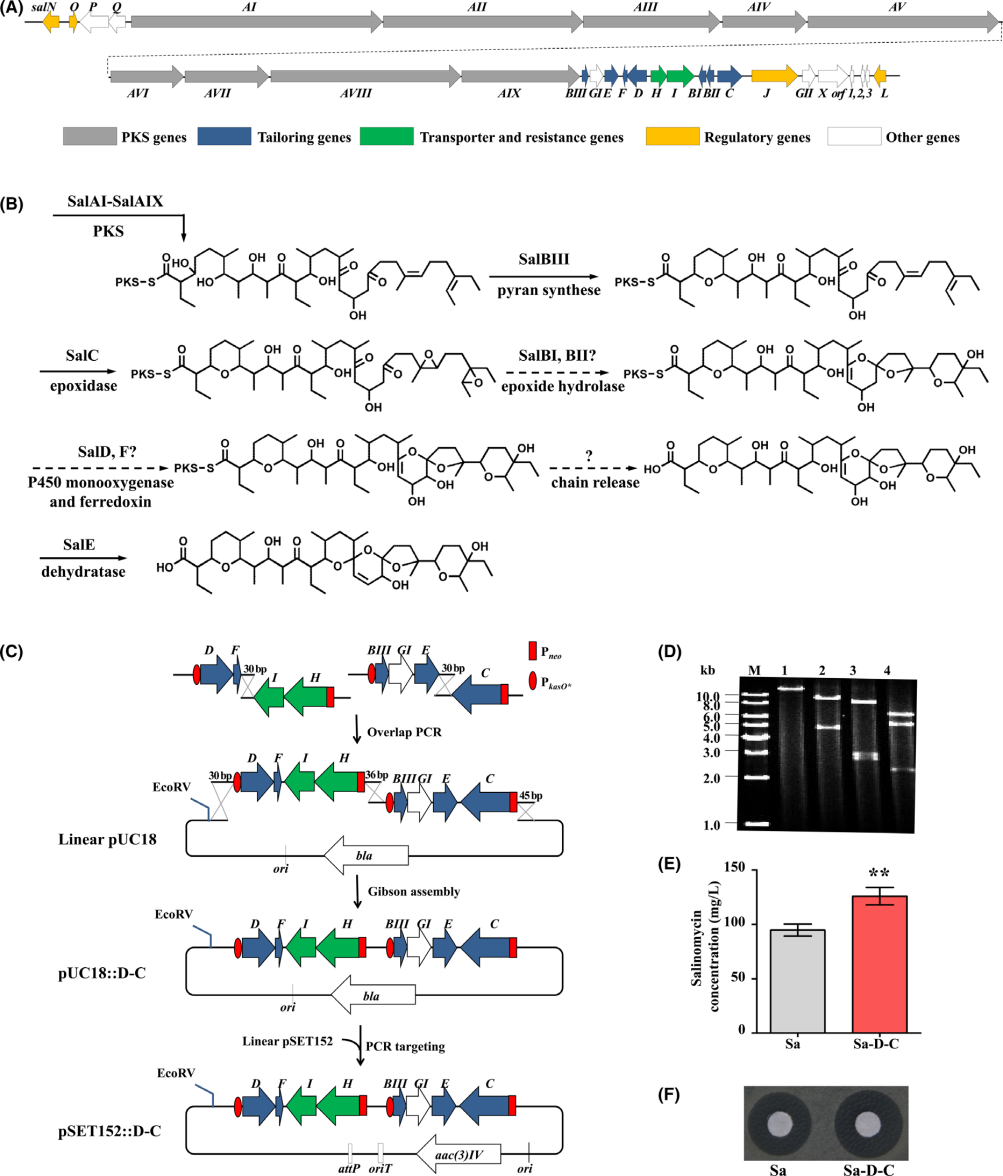
Effect of the overexpression of key gene modules on salinomycin production. A. Genetic organization of salinomycin biosynthetic gene cluster in *S. albus*. B. Proposed pathway of salinomycin biosynthesis. C. Construction of recombinant plasmid pSET152::D‐C, an assembly of *salBIII‐E*, *salC*, *salD‐F* and *salH‐I* (abbreviated as *salD‐C*) on pSET152. D. Agarose gel electrophoresis of restriction fragments from plasmid pSET152::D‐C. M, 1 kb DNA ladder; lanes 1‐4, plasmid pSET152::D‐C digested with EcoRV (14 415 bp), KpnI (9522 bp, 4893 bp), BamHI (8605 bp, 3005 bp, 2805 bp) and PvuII (6607 bp, 5394 bp, 2414 bp) respectively. The expected sizes of DNA fragments are shown in brackets above. E. HPLC analysis of salinomycin production in the fermentation broth of Sa and Sa‐D‐C strains. Data are presented as the averages of three independent experiments. Error bars indicate standard deviations (SD). Significant difference between the recombinant strains and wild‐type strain (Sa) was confirmed by Student’s *t*‐test (** represents *P* < 0.01). F. Bioassays of salinomycin from the fermentation broth of Sa and Sa‐D‐C strains against *B. cereus*. Sa, *S. albus* CGMCC 4.5716 (wild‐type strain); Sa‐D‐C, *salD‐C* overexpression strain.

Salinomycin biosynthetic gene cluster (*sal*) consists of at least 27 genes (Fig. 2A). In order to enhance salinomycin production, an alternative strategy of reconstructing a minimized *sal* cluster was proposed. In this strategy, the tailoring and resistance genes were primarily selected for the construction, which would be ligated together via Gibson assembly. Based on the proposed salinomycin biosynthetic pathway (Fig. 2B) (Jiang *et al*., 2012; Yurkovich *et al*., 2012; Jiang *et al*., 2015; Luhavaya *et al*., 2015), the epoxidase gene *salC*, epoxide hydrolase genes *salBI* and *salBII*, dehydratase gene *salE*, furan synthase gene *salBIII*, P450 monooxygenase gene *salD* and the cognate ferredoxin gene *salF* as well as resistance genes *salH* and *salI* were selected for evaluation. The transcriptional units (or gene modules) containing tailoring or resistance genes *salBIII‐E, salD‐F*, *salH‐I*, *salBII‐BI* and *salC* were overexpressed under the control of P_kasO*_ or P*_neo_* promoter respectively. The resulting constructs were introduced individually into *S. albus* CGMCC 4.5716 through conjugal transfer to generate corresponding recombinant strains (Sa‐neoBIII‐E, Sa‐kasO*BIII‐E, Sa‐neoD‐F, Sa‐kasO*D‐F, Sa‐neoH‐I, Sa‐kasO*H‐I, Sa‐neoBII‐BI, Sa‐kasO*BII‐BI, Sa‐neoC and Sa‐kasO*C), which were verified by PCR amplification (Fig. S1A). Salinomycin production of Sa‐kasO*BIII‐E, Sa‐kasO*D‐F, Sa‐neoD‐F, Sa‐neoC and Sa‐kasO*C strains was 13%‐23% higher than that of *S. albus* CGMCC 4.5716, whereas no obvious yield improvement was observed for other strains (Fig. S1B). The results suggested that overexpression of *salBIII‐E, salD‐F* and *salC* gene modules are significant for increasing salinomycin production in *S. albus* CGMCC 4.5716.

In order to achieve integrative effects, aforesaid gene modules involved in salinomycin overproduction were combined via Gibson assembly, including modules P_kasO*_::*salBIII‐E*, P*_neo_*::*salC*, P_kasO*_::*salD‐F* as well as resistance genes *salH* and *salI* to generate a pSET152‐based recombinant plasmid (Fig. 2C), which was further confirmed by EcoRV, KpnI, BamHI and PvuII digestions and designated as pSET152::D‐C (Fig. 2D). Then, it was introduced into *S. albus* CGMCC 4.5716 through conjugal transfer to generate recombinant strain Sa‐D‐C. HPLC analysis revealed that salinomycin production from the fermentation broth of Sa‐D‐C increased by 30% compared with that of wild‐type strain, indicating that overexpressing the construct combining structural and tailoring genes is more efficient than single gene module, and the bioassays against *B. cereus* CGMCC were consistent with HPLC analysis (Fig. 2E and F). It was implied that integrating gene modules to superimpose their genetic merits would be a feasible route for further improving the production of salinomycin.

### Construction of a salinomycin biosynthetic mini‐cluster

**Fig. 3:**
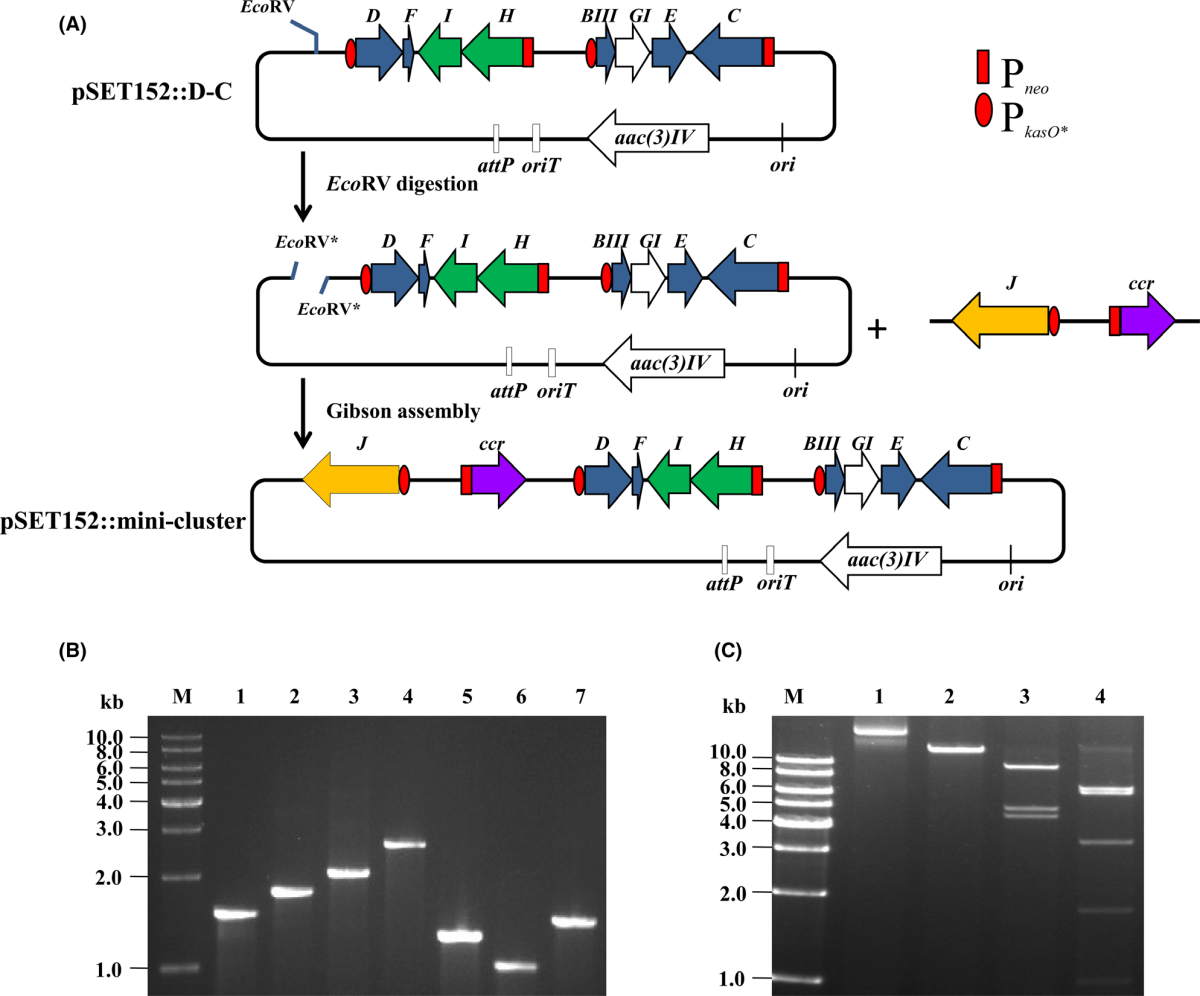
Construction and verification of the recombinant plasmid pSET152::mini‐cluster. A. Construction of the recombinant plasmid pSET152::mini‐cluster. B. Agarose gel electrophoresis of PCR amplification products from the plasmid pSET152::mini‐cluster. M, 1 kb DNA ladder; lanes 1–7, PCR products using primer pairs MCS‐F/qJ‐F (1491 bp), salJ‐in‐R/qccr‐R (1772 bp), ccr‐F/qD‐R (2056 bp), qD‐F/qT1‐F (2631 bp), qH‐R/qBI‐R (1269 bp), salE‐in‐F/qC‐F (1010 bp) or qC‐R/MCS–R (1431 bp) respectively. C. Agarose gel electrophoresis of restriction fragments from the plasmid pSET152::mini‐cluster. M, 1 kb DNA ladder; lane 1, the recombinant plasmid pSET152::mini‐cluster; lanes 2–4, the restriction fragments of plasmid pSET152::mini‐cluster digested with EcoRI (18 852 bp), EcoRI + KpnI (9529 bp, 4893 bp, 4430 bp) or NcoI (6553 bp, 6170 bp, 3306 bp, 1788 bp, 1035 bp) respectively. The expected sizes of DNA fragments are shown in brackets above.

Based on the plasmid pSET152::D‐C, more biosynthetic elements were considered to further intensify the strain potential for producing salinomycin. Looking into the *sal* gene cluster, tailoring genes situated in plasmid pSET152::D‐C are 8.58 kb in size, while *pks* genes indispensable for the polyketide chain biosynthesis are more than 78 kb. How to enhance *pks* transcription is of great importance but the challenge is that these genes are too large to manipulate easily. A shortcut strategy to circumvent cloning the whole sequence of *sal* or *pks* was proposed. As mentioned above, overexpression of *salJ* under the control of P_kasO*_ promoter increased the production of salinomycin by 95%, and also, it was demonstrated that SalJ can activate the transcription of *pks* genes *salAI*‐*AIX* and other operons by binding to their promoter regions (Zhu *et al*., 2016). Thus, indirectly upregulating the transcription level of *pks* genes by overexpressing *salJ* was employed. On the other hand, because secondary metabolite biosynthesis relies on various precursors supplied from other pathways, increasing the precursor pools would be one way to break through the bottleneck of antibiotic production. Among those, a discrete gene *ccr* (encoding crotonyl‐CoA reductase) situated outside of the *sal* cluster on chromosome plays crucial roles in ethylmalonyl‐CoA biosynthesis, which is a key precursor of salinomycin biosynthesis (Lu *et al*., 2016; Zhang *et al*., 2017a, 2019). Increasing ethylmalonyl‐CoA supply is particularly essential while other structural genes are overexpressed. Therefore, two more genes *salJ* and *ccr* were considered for further integrating into the plasmid pSET152::D‐C to generate a mini‐cluster for high production of salinomycin. The P*_neo_*::*ccr* and P_kasO*_::*salJ* were combined to generate *salJ*‐*ccr* cassette by overlap extension PCR, which was then ligated with linear pSET152::D‐C by Gibson assembly to generate pSET152::mini‐cluster (Fig. 3A). The resulting plasmid was verified by PCR amplification and restriction digestions (Fig. 3B and C).

### Identification of a chassis strain favourable for salinomycin production

To effectively express the mini‐cluster, it is essential to choose proper chassis host. In our previous work, a streptomycin‐resistant mutant of *S. albus* CGMCC 4.5716 (Str‐99) with higher salinomycin production was obtained by ribosome engineering, in which a truncated RsmG was identified (Li *et al*., 2019b).

**Fig. 4:**
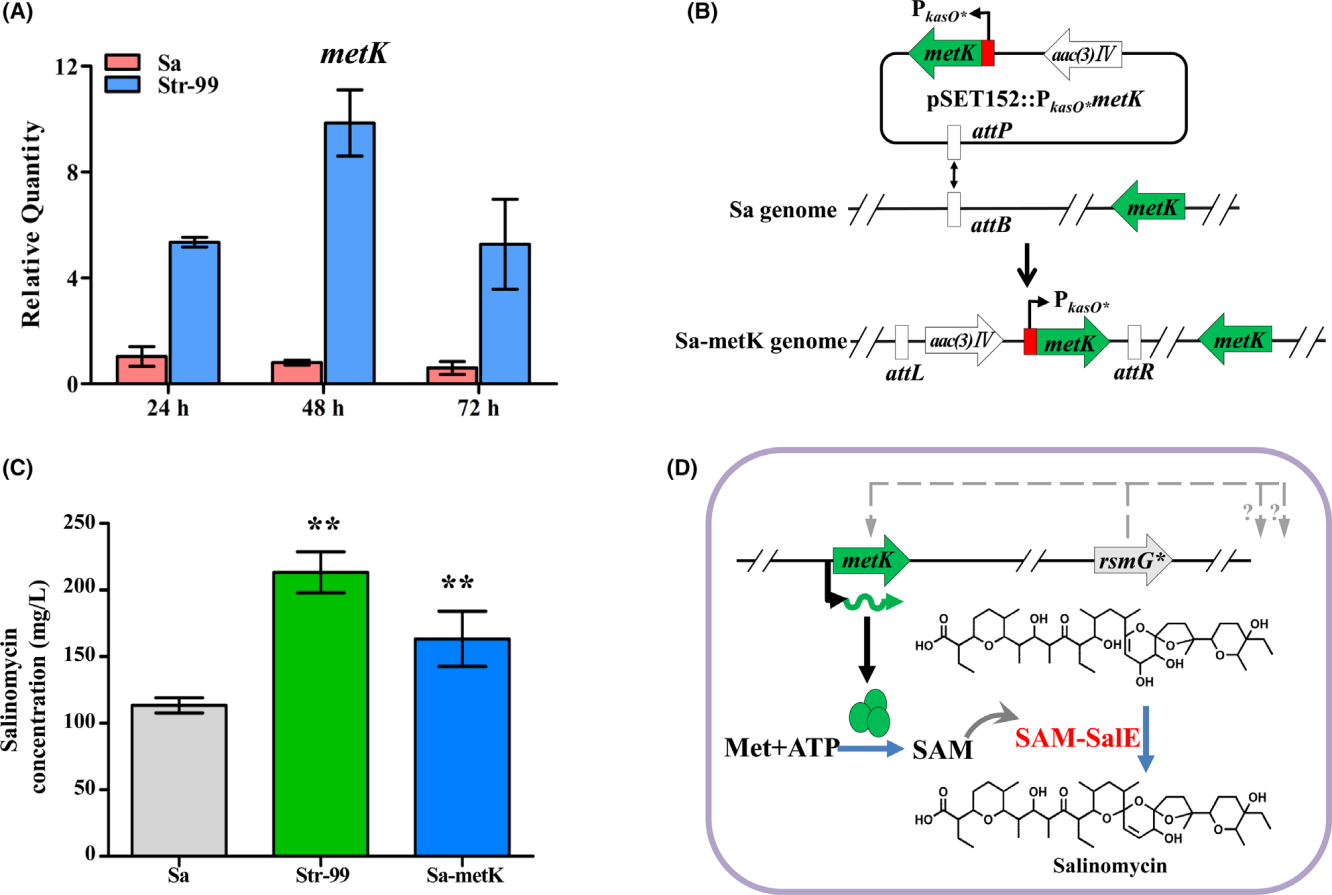
Effect of *metK* overexpression on salinomycin production. A. Transcriptional analysis of *metK* in Sa and Str‐99 strains. The transcriptional level of *metK* was normalized internally to that of *hrdB* transcription of Sa. Data are presented as the averages of three independent experiments. Error bars indicate standard deviations (SD). B. Construction of *metK* overexpression strains. C. HPLC analysis of salinomycin production from the fermentation broth of Sa, Str‐99 and Sa‐metK strains. Significant difference between the recombinant strains and wild‐type strain (Sa) was confirmed by Student’s *t*‐test (** represents *P* < 0.01). D. Proposed pleiotropic effect of *rsmG* mutation and *metK* overexpression on salinomycin production. Sa, *S. albus* CGMCC 4.5716 (wild‐type strain); Str‐99, the streptomycin‐resistant mutant of *S. albus* CGMCC 4.5716; Sa‐metK, *metK* overexpression strain; rsmG*, mutated *rsmG* gene with a base C insertion at position 26.

In order to know whether the resulting Str‐99 can be used as a better chassis host than the wild‐type strain for further enhancing salinomycin production, more assessments were performed. It has been reported that the expression of SAM synthetase MetK was increased in RsmG mutants of several *Streptomyces* strains (Nishimura *et al*., 2007; Tanaka *et al*., 2009), whereas SAM is a co‐factor of SalE that catalyses the last step of the salinomycin synthetic pathway (Jiang *et al*., 2015). It is intriguing to know whether the transcription of *metK* is upregulated in Str‐99, and how it correlates with salinomycin production. BlastP analysis revealed that MetK of *S. albus* CGMCC 4.5716 shares high sequence identity (> 93%) with those of other *Streptomyces* species (Fig. S2). RT‐qPCR analysis showed that the transcriptional level of *metK* was remarkably increased in Str‐99 compared to that in the wild‐type strain (Fig. 4A). Salinomycin production in Sa‐metK was also increased by 45% (Fig. 4B and C), suggesting that MetK overexpression is beneficial for salinomycin biosynthesis.

Since RsmG is involved in the methylation of 16S rRNA (Nishimura *et al*., 2007), *rsmG* mutation and *metK* overexpression in Str‐99 may have pleiotropic effects on cells favourable for enhancing salinomycin production (Fig. 4D). So Str‐99 would be a choice of suitable chassis host. A compatible host strain to enable the accommodation of the modified pathways is critical for improving antibiotics production. For well‐established biosynthetic pathways, knocking out the redundant genes and competitive gene clusters or introducing precursor biosynthetic pathways in amenable strains proved successful for generating chassis hosts (Zhang *et al*., 2017a). Otherwise, identifying the optimal strains using high‐throughput screening platform from numerous spontaneous mutants was acceptable. Here, we confirmed that ribosome engineering applied in directed screening of mutants for high production of antibiotics (Ochi, 2007) is worthy of development and application.

### Effect of mini‐cluster expression on salinomycin production

**Fig. 5:**
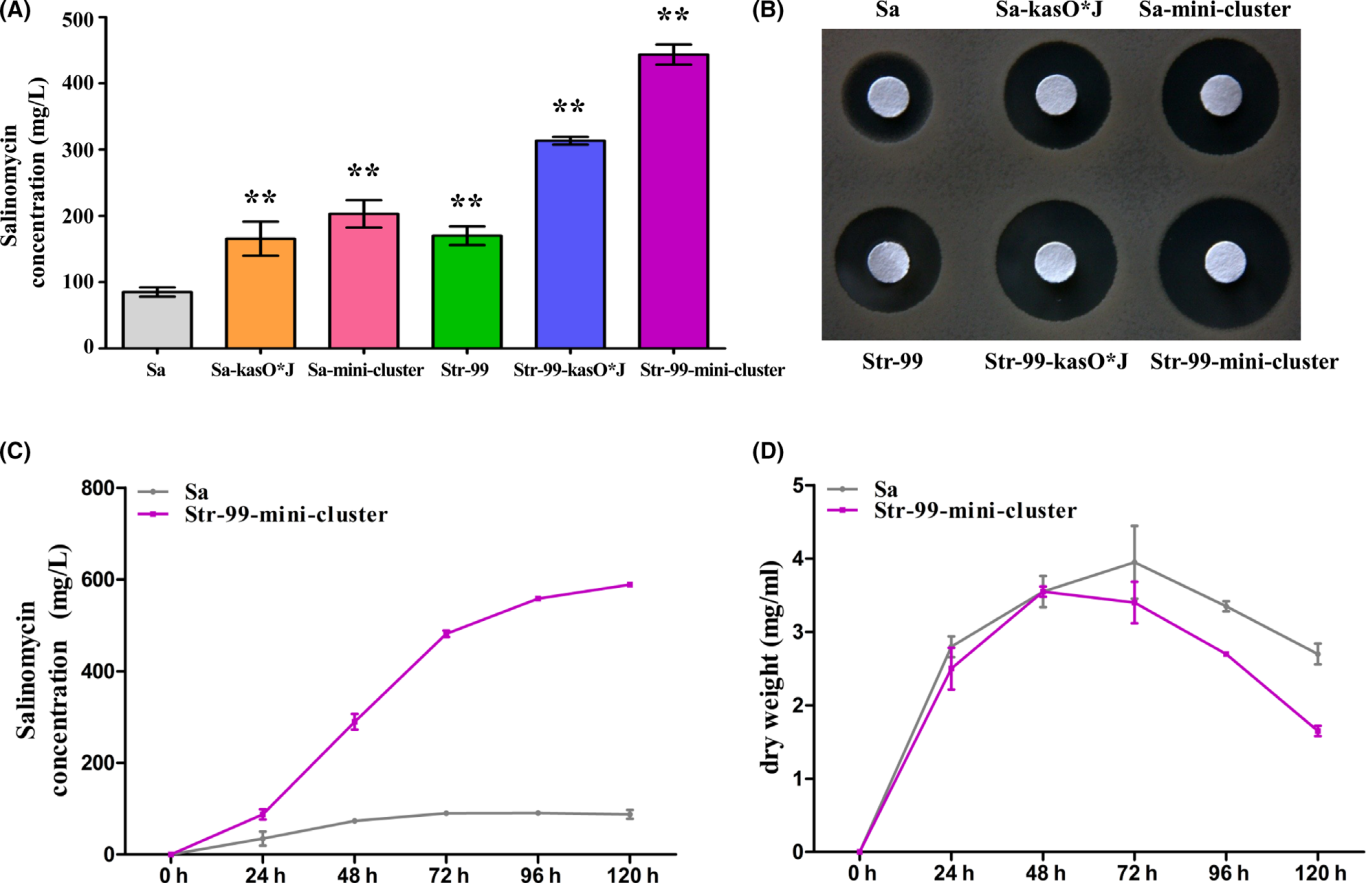
Effect of the overexpression of mini‐cluster on salinomycin production. a whole gene cluster in a defineda whole gene cluster in a defined A. HPLC analyses of salinomycin production from the fermentation broth of different strains. Data are presented as the averages of three independent experiments. Error bars indicate standard deviations (SD). Significant difference between the recombinant strains and wild‐type strain (Sa) was confirmed by Student’s *t*‐test (** represents *P* < 0.01). a whole gene cluster in a defined B. Bioassays of salinomycin from the fermentation broth of different strains against *B. cereus*. a whole gene cluster in a defined C. Time‐course of salinomycin production in Sa and Str‐99‐mini‐cluster determined by HPLC. a whole gene cluster in a defined D. Growth curves of strains Sa and Str‐99‐mini‐cluster in liquid culture. Sa, *S. albus* CGMCC 4.5716 (wild‐type strain). Str‐99, a streptomycin‐resistant mutant of *S. albus* CGMCC 4.5716. Sa‐kasO*J and Str‐99‐kasO*J, Sa or Str‐99 contains *salJ* under the control of P_kasO*_ promoter for overexpression respectively. Sa‐mini‐cluster and Str‐99‐mini‐cluster, Sa or Str‐99 contains mini‐cluster respectively.

In order to evaluate the effect of mini‐cluster expression on salinomycin production, the plasmid pSET152::mini‐cluster was introduced into *S. albus* CGMCC 4.5716 by conjugal transfer to obtain recombinant strain Sa‐mini‐cluster. Salinomycin production of *S. albus* CGMCC 4.5716 and its derivative strains was detected and quantified by HPLC analysis and bioassays against *B. cereus*. As shown in Fig. 5A, the production of salinomycin in Sa‐mini‐cluster was 2.3‐fold of that in *S. albus* CGMCC 4.5716. Compared to the yield increment in Sa‐D‐C (Fig. 2E), the combinatorial mini‐cluster more efficiently enhanced salinomycin production. Next, we would like to know the host contribution to salinomycin production. Str‐99 mentioned above was assessed as a potential chassis strain. The pSET152::mini‐cluster or pSET152::P_kasO*_ J was introduced into Str‐99, resulting recombinant strains Str‐99‐mini‐cluster and Str‐99‐kasO*J respectively. Salinomycin production in Str‐99‐mini‐cluster was 2.6‐fold of that in Str‐99 and 5.1‐fold of wild‐type strain *S. albus* CGMCC 4.5716; it also increased 2.2‐fold in comparison with Sa‐mini‐cluster, the wild‐type strain harbouring *sal* mini‐cluster. Meanwhile, *salJ* overexpression in Str‐99‐kasO*J led to 1.9‐fold increase of salinomycin production compared with that in Sa‐kasO*J. The bioactivity of the fermentation broth from these strains was consistent with HPLC analysis (Fig. 5B). These results revealed that a compatible host strain is essential, and combining mini‐cluster and ribosome engineering could achieve significant superposition effect on product improvement.

Further analyses on salinomycin production and growth rate of the wild‐type strain and engineering strain Str‐99‐mini‐cluster were performed (Fig. 5C and D). The production of salinomycin in Str‐99‐mini‐cluster was consistently higher at all the tested time points (24 ˜ 120 h), while the growth trend in both strains kept close, except for the fact that the biomass of Str‐99‐mini‐cluster at 96 h and 120 h was getting lower than that of Sa probably due to nutrients exhausting in the medium. Nevertheless, it was convinced that the ready‐to‐use mini‐cluster construct would be applicable in more suitable hosts.

**Fig. 6:**
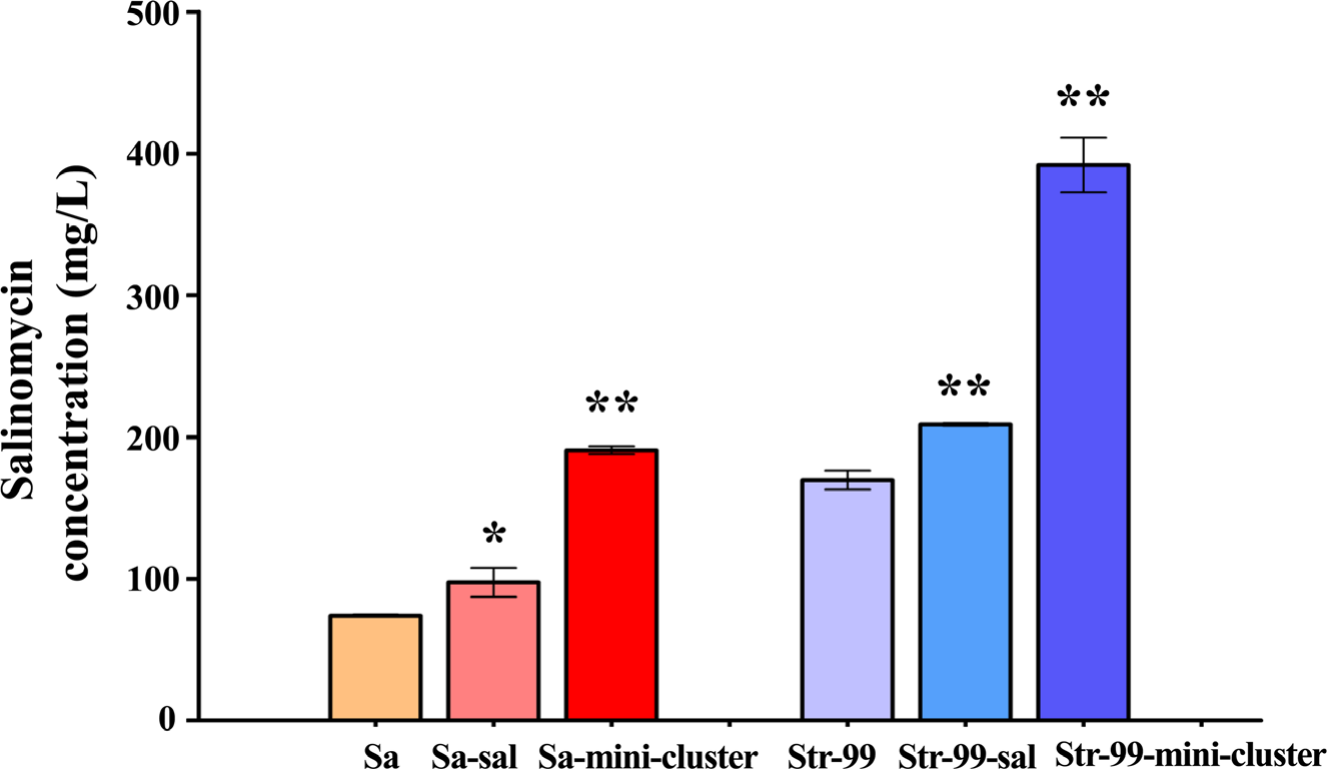
Effect of the overexpression of *sal* gene cluster on salinomycin production. Sa, *S. albus* CGMCC 4.5716 (wild‐type strain). Str‐99, a streptomycin‐resistant mutant of *S. albus* CGMCC 4.5716. Sa‐sal and Str‐99‐sal, Sa or Str‐99 contains *sal* gene cluster respectively. Sa‐mini‐cluster and Str‐99‐mini‐cluster, Sa or Str‐99 contains mini‐cluster respectively. Error bars indicate standard deviations (SD). Significant difference between the recombinant strains and Sa or Str‐99 was confirmed by Student’s *t*‐test (* and ** represent *P* < 0.05 and *P* < 0.01 respectively).

Interestingly, there was only 20%–30% increase in the whole *sal* gene cluster overexpression strains Sa‐sal and Str‐99‐sal, which were generated by conjugal transfer of plasmid pBAC::sal into Sa and Str‐99 respectively (Fig. 6). Thus, it was concluded that the mini‐cluster showed much higher efficiency than the whole *sal* gene cluster. It is not unusual that the whole gene cluster amplification could not result in sufficient efficiency as anticipated. The definitive reasons for this are not absolutely clear yet, but general envisagement could be the stability of gene clusters, aggravated cellular metabolism burden or some kind of 'immune mechanism' of cells. In contrast, the genes in mini‐cluster were rather directly enhanced since multiple strong promoters were accommodated.

Recently, various technologies of pathway engineering have emerged, such as targeting rate‐limiting steps or engineering and amplification of whole gene clusters, but reconstitution of a combinatorial mini‐cluster could be an important alternative. This ingenious design can easily perform genetic manipulations on not only the cluster‐situated genes, but also the genes out of the cluster if necessary. Furthermore, compared to the original gene cluster (104 kb), *sal* mini‐cluster size (13 kb) is considerably smaller, so the technical obstacles of manipulating large DNA fragments could be bypassed. It is also noteworthy that strong regulatory genes encoding crucial activators of the massive *pks* or *nrps* gene operons are central for constructing the mini‐cluster and minimizing its size. They are present in many strains producing industrially important antibiotics, such as *aveR* for avermectin (Guo *et al*., 2010), *rapG* and *rapH* for rapamycin (Kuscer *et al*., 2007) and *depR1* for daptomycin biosynthesis (Yuan *et al*., 2016), indicating that similar mini‐cluster strategy could be adopted. Taken together, devising a mini‐cluster towards manipulating multiple pathways can exert actions as a whole gene cluster in a defined genetic context, which provided a universal approach for engineering the massive polyketide gene clusters.

Due to the large capacity harbouring various functional modules, broader application perspective of mini‐cluster is perceived. In the past years, synthetic biology has been advanced magnificently including the creation of new species (Gibson *et al*., 2010), and discovery of numerous artificial genetic elements, providing valuable resources for mini‐cluster construction to expand their functions. Among them, modulating the production profile of the antibiotics by synthetic regulatory circuits proved to be feasible, which is particularly critical for hazardous products (Liang *et al*., 2020). On the other hand, modular replacement of key structural genes via combinatorial biosynthesis was employed to create novel structural compounds derived from different microbial resources. As we reported previously, a hybrid antibiotic polynik A was generated by combining the genes responsible for the biosynthesis of nucleoside moiety from *Streptomyces ansochromogenes* and peptidyl moiety from *Streptomyces cacaoi* (Li *et al*., 2011). For polyketide antibiotics, *pks* genes or tailoring genes are generally chosen for modular replacement. We are confident that mini‐cluster could selectively incorporate different functional elements for broad applications.

### Transcriptional analysis of representative genes for salinomycin biosynthesis

**Fig. 7:**
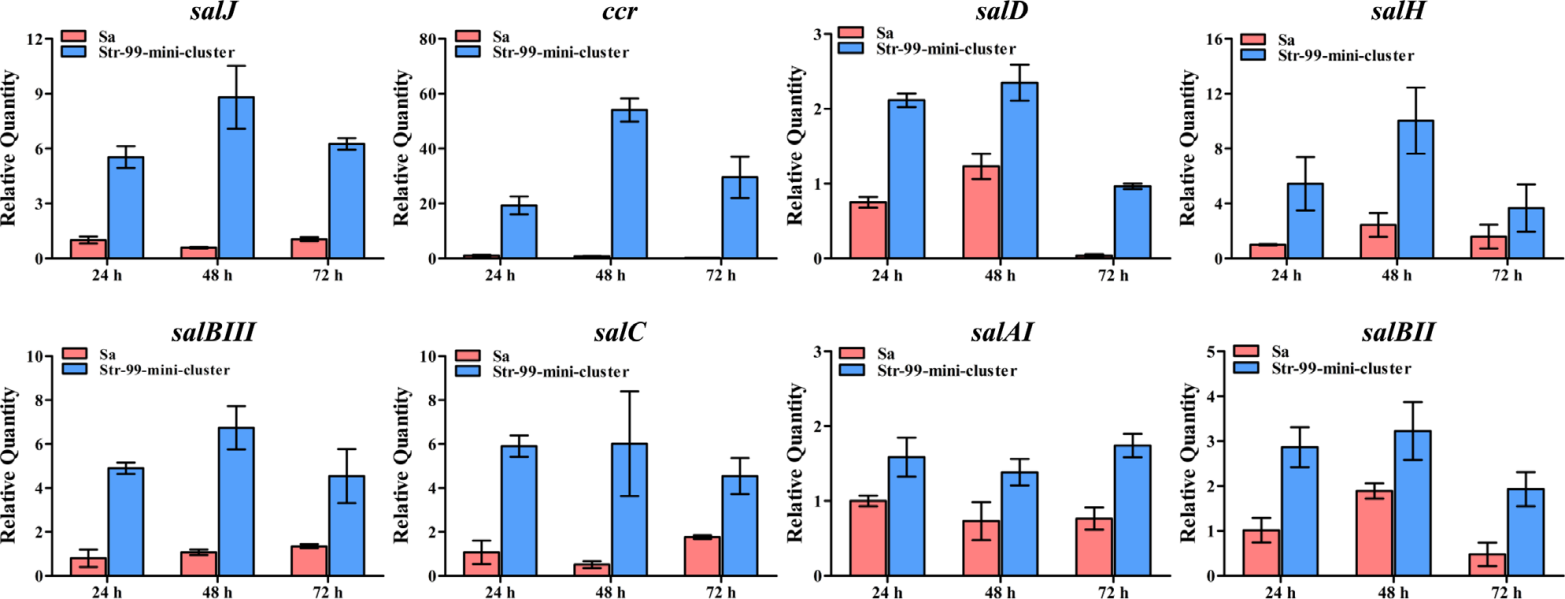
RT‐qPCR transcriptional analysis of salinomycin biosynthetic genes in Sa and Str‐99‐mini‐cluster strains. Sa, *S. albus* CGMCC 4.5716 (wild‐type strain). Str‐99‐mini‐cluster strain, Str‐99 strain contains the mini‐cluster for overexpression. Error bars show standard deviations (SD). Transcriptional levels of the genes were normalized internally to the level of *hrdB* transcription. Data are presented as means ± standard deviations (SD) of three independent experiments.

To determine the transcriptional profiles of key genes for salinomycin production in the strains containing mini‐cluster, RT‐qPCR analysis was performed. Total RNAs of *S. albus* CGMCC 4.5716 and Str‐99‐mini‐cluster were isolated from the mycelia grown at different time points (24, 48 and 72 h), and RT‐qPCR analysis was conducted. The transcription of all tested genes of Str‐99‐mini‐cluster was significantly upregulated in comparison with that of the wild‐type strain (Fig. 7). For *salAI* and *salBII* genes situated on chromosome, nearly 2‐fold increase was observed at 72 h due to positive regulation of SalJ. The transcription of genes included in mini‐cluster markedly increased at 24 h and maintained at a high level over the tested period (72 h). The transcription of *ccr* in Str‐99‐mini‐cluster strain increased to nearly 60‐fold in comparison with that in the wild‐type strain. These data corroborated that the mini‐cluster excluding *pks* genes could exert its function as that of whole *sal* cluster for salinomycin biosynthesis in a suitable producing host strain.

**Fig. 8:**
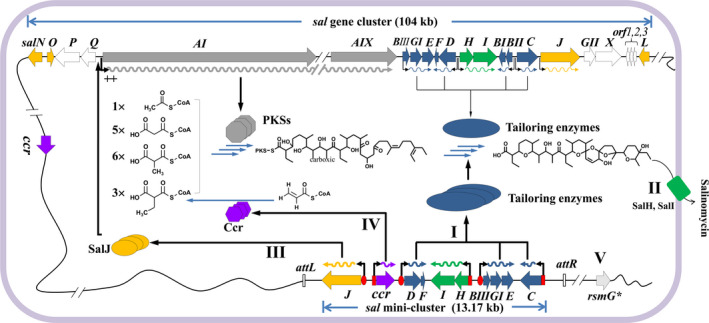
Overview of *sal* mini‐cluster in Str‐99 chassis strain for promoting salinomycin production. This combinatorial optimization strategy consists of five routes. Route **I** involves the overexpression of tailoring genes driven by strong promoters (P_kasO*_ or P*_neo_*). Route **II** involves the overexpression of transporter genes (*salH*, *salI*) to enhance the tolerance of salinomycin. Route **III** involves the overexpression of the activator gene *salJ* to upregulate the transcription of *pks* operon. Route **IV** involves the overexpression of *ccr* (crotonyl‐CoA reductase gene) to improve the formation of ethylmalonyl‐CoA precursor from crotonyl‐CoA. Route **V** involves the acquirement of a host strain Str‐99 with *rsmG* mutation (here represented as rsmG*) by ribosome engineering for salinomycin production.

In summary, a novel strategy of modular assembly combined with ribosome engineering was developed to improve salinomycin production (Fig. 8). A combinatorial 'mini‐*sal* gene cluster' was constructed (route **I**, **II**, **III**, **IV**). The *pks* operon was not included in the mini‐cluster, but it was upregulated by the overexpression of SalJ activator encoded by *salJ*, a cluster‐situated regulator (CSR) (route **III**). Meanwhile, the mutant strain Str‐99 obtained by ribosome engineering was used as a chassis host (route **V**). The yield of salinomycin in Str‐99‐mini‐cluster was 5.1‐fold of that in the wild‐type strain. Also, mini‐cluster showed much higher efficiency than whole *sal* gene cluster, demonstrating its potential for wider application. This approach may be extended to other polyketide bioactive molecules, and mini‐cluster set up a basis for generating diverse derivatives of salinomycin for further lead optimization.

## Experimental procedures

### Bacterial strains, plasmids, primers and growth conditions

The strains and plasmids used in this study are listed in Table S1. *S. albus* CGMCC 4.5716 and its derivatives were grown on ISP4 medium (Difco™, BD) for growth and spore formation, and TSBY medium (3% TSB, 10.3% sucrose and 1% yeast extract) for isolation of the total DNA and also for seed culture; YMG (0.4% yeast extract, 1% malt extract and 0.4% glucose) was used as the fermentation medium. When necessary, the final concentrations of antibiotics used were as follows: for selection of *E. coli* transformants, ampicillin or apramycin, 100 μg ml^−1^ in LB; for selection of *Streptomyces* exconjugants, apramycin, 5 μg ml^−1^ in ISP4 or TSBY; nalidixic acid, 25 μg ml^−1^ in ISP4.

### DNA manipulation, conjugation and transcriptional analysis

DNA isolation and manipulation in *Streptomyces* and *E. coli* were carried out as described previously (Kieser *et al*., 2000; Sambrook and Russell, 2001). PCR targeting was performed as described by Gust *et al*. (2003). Gibson assembly was conducted following the procedures as shown in the one‐step isothermal DNA assembly protocol (Gibson *et al*., 2009). Intergeneric conjugation between *E. coli* ET12567/pUZ8002 and *S. albus* was performed according to the standard protocols (Kieser *et al*., 2000). RNA isolation and real‐time quantitative PCR (RT‐qPCR) were performed as described previously (Li *et al*., 2016).

### Construction of recombinant plasmids and strains for overexpression of corresponding gene modules

The primers are listed in Table S2. To obtain the recombinant plasmids (pSET152::P*_hrdB_* J, pSET152::P*_neo_* J, pSET152::P_kasO*_ J, pSET152::P*_neo_* BIII‐E, pSET152::P_kasO*_ BIII‐E, pSET152::P*_neo_* D‐F, pSET152::P_kasO*_ D‐F, pSET152::P*_neo_* H‐I, pSET152::P_kasO*_ H‐I, pSET152::P*_neo_* C, pSET152::P_kasO*_ C, pSET152::P*_neo_* BII‐BI, pSET152::P_kasO*_ BII‐BI and pSET152::P_kasO*_ metK), the promoter and target module were inserted into the pSET152 vector through double enzymatic digestion and ligation, and the resulting plasmids were verified by sequencing (Invitrogen Corporation，Beijing, China). The plasmids were introduced into *S. albus* CGMCC 4.5716 by conjugation transfer, and then, the corresponding engineered strains were screened from the exconjugants on ISP4 medium containing apramycin and nalidixic acid.

### Construction of pSET152::D‐C plasmid

Construction of pSET152::D‐C was conducted in two steps: step 1 was to assemble the required gene modules using cloning vector pUC18; step 2 was to replace the pUC18 DNA sequence of the construct with that of pSET152 for integrative overexpression in *Streptomyces*. Specifically, P_kasO*_::*salD‐F* and P*_neo_*::*salH‐I* modules were amplified with primer pair MC‐DF‐F/MC‐DF‐R and MC‐HI‐F/MC‐HI‐R using plasmid pSET152::P_kasO*_ D‐F and pSET152::P*_neo_* H‐I as the template. The two modules were ligated by overlap extension PCR using primer pair MC‐DF‐F/MC‐HI‐R to generate a 4487 bp of *salD‐F‐H‐I* cassette. *salBIII‐E‐C* cassette was generated using the similar method. At the same time, a DNA fragment of pUC18 vector was obtained by two‐step PCR amplification using primer pair pUC18‐F/pUC18‐R for the first round and pUC18‐F/pUC18‐R2 for the second round to introduce an EcoRV restriction site and the overlapped sequence for assembly with *salD*. Subsequently, it was ligated with *salD‐F‐H‐I* and *salBIII‐E‐C* cassettes via Gibson assembly to generate plasmid pUC18::D‐C. Finally, linear DNA sequence of pSET152 was generated by PCR using primer pair Targeting‐152‐F/Targeting‐152‐R and then was electroporated into *E. coli* BW25113/pIJ790 containing pUC18::D‐C to replace the pUC18 vector fragment by λ‐Red‐mediated PCR targeting. The resulting plasmid was designated as pSET152::D‐C.

### Construction of recombinant plasmid pSET152::mini‐cluster

In order to construct the recombinant plasmid pSET152::mini‐cluster, P*_neo_* promoter was amplified using primer pair ccr‐Pneo‐F/ccr‐Pneo‐R from pUC119::*neo*, and *ccr* gene was amplified using primer pair MC‐ccr‐F/MC‐ccr‐R with genomic DNA of *S. albus* CGMCC 4.5716 as template. Two DNA fragments were ligated by overlap extension PCR with primer pair ccr‐Pneo‐F/MC‐ccr‐R to generate module P*_neo_*::*ccr*. Module P_kasO*_::*salJ* amplified from the plasmid pSET152::P_kasO*_ J using primer pair MC‐J‐F/MC‐J‐R was then combined with P*_neo_*::*ccr* by overlap extension PCR with primer pair MC‐J‐R/MC‐ccr‐R to generate 4581 bp *salJ*‐*ccr* cassette. The resulting cassette and linear pSET152::D‐C obtained by EcoRV digestion were further ligated via Gibson assembly to generate pSET152::mini‐cluster.

### Bioassay and HPLC analysis of salinomycin production

Salinomycin produced by *S. albu*s and its derivatives was evaluated using a disc diffusion method against *Bacillus cereus* as an indicator strain (Table S1). HPLC analysis was performed as described previously (Li *et al*., 2019b).

## Conflict of interest

The authors declare that they have no competing interests.

## Supporting information


**Table S1** Strains and plasmids used in this study
**Table S2** Primers used in this study
**Fig. S1.** PCR analysis of the gene modules in the overexpression strains (A) and the HPLC analysis of salinomycin production (B). (A) M, DNA ladder; Lanes 1, 4, 7, 10, 13, 16, 19, 22, 25 and 28: negative controls; Lanes 2‐3, 5‐6, 8‐9, 11‐12, 14‐15, 17‐18, 20‐21, 23‐24, 26‐27 and 29‐30: PCR products using genomic DNAs of strains Sa‐kasO*BIII‐E, Sa‐neoBIII‐E, Sa‐kasO*D‐F, Sa‐neoD‐F, Sa‐kasO*H‐I, Sa‐neoH‐I, Sa‐kasO*BII‐BI, Sa‐neoBII‐BI, Sa‐kasO*C and Sa‐neoC as template, respectively.
**Fig. S2.** Sequence alignment of MetK from different *Streptomyces* (Sc, *Streptomyces coelicolor*. Ss, *Streptomyces* *spectabilis*. Sl, *Streptomyces* *lividans*. Sa, *Streptomyces albus*).
