# Supplementary material for: Reconstitution of a mini‐gene cluster combined with ribosome engineering led to effective enhancement of salinomycin production in Streptomyces albus
Source: Microb Biotechnol. 2020 Dec 3;14(6):2356–68. doi: 10.1111/1751-7915.13686 (PMC8601195; doi:10.1111/1751-7915.13686)
Supplement: Supplementary file 1 — Table S1 Strains and plasmids used in this study Table S2 Primers used in this study Fig. S1. PCR analysis of the gene modules in the overexpression strains (A) and the HPLC analysis of salinomycin production (B). (A) M, DNA ladder; Lanes 1, 4, 7, 10, 13, 16, 19, 22, 25 and 28: negative controls; Lanes 2‐3, 5‐6, 8‐9, 11‐12, 14‐15, 17‐18, 20‐21, 23‐24, 26‐27 and 29‐30: PCR products using genomic DNAs of strains Sa‐kasO*BIII‐E, Sa‐neoBIII‐E, Sa‐kasO*D‐F, Sa‐neoD‐F, Sa‐kasO*H‐I, Sa‐neoH‐I, Sa‐kasO*BII‐BI, Sa‐neoBII‐BI, Sa‐kasO*C and Sa‐neoC as template, respectively. Fig. S2. Sequence alignment of MetK from different Streptomyces (Sc, Streptomyces coelicolor. Ss, Streptomyces spectabilis. Sl, Streptomyces lividans. Sa, Streptomyces albus). [file MBT2-14-2356-s001.docx]

**Reconstitution of a mini-gene cluster combined with ribosome engineering led to effective enhancement of salinomycin production in *Streptomyces albus***

**Dong Li^†,a,b^ Yuqing Tian^†,a^ Xiang Liu^a,b^ Wenxi Wang^a,b^ Yue Li^a^ Huarong Tan^*,a,b^ Jihui Zhang^*,a^**

*^a^ State Key Laboratory of Microbial Resources, Institute of Microbiology, Chinese Academy of Sciences, Beijing 100101, China*；

*^b^ College of Life Sciences, University of Chinese Academy of Sciences, Beijing 100049, China*

**^†^: These authors contributed equally to this work.**

***: Corresponding authors (Jihui Zhang, email: zhang.jihui@im.ac.cn, Tel: +86-10‐64807467; Huarong Tan, email: tanhr@im.ac.cn, Tel/Fax: +86-10-64807461)**

**Running title:**

**Mini-cluster for enhancing salinomycin production**

**Table S1** Strains and plasmids used in this study

| **Strains/plasmids** | | | **Genotype/description** | | **Reference/source** | | | | |
| --- | --- | --- | --- | --- | --- | --- | --- | --- | --- |
| ***Streptomyces albus*** | | |  | | | |  | | |
| CGMCC 4.5716 | | | Wild-type strain | | | | CGMCC | | |
| Str-99 | | | A streptomycin-resistant mutant from *S. albus* CGMCC 4.5716 | | | | Li *et al*., 2019 | | |
| Sa-metK | | | *S. albus* CGMCC 4.5716 containing pSET152::P*_kasO*_*metK | | | | This study | | |
| Sa-hrdBJ | | | *S. albus* CGMCC 4.5716 containing pSET152::P*_hrdB_*J | | | | This study | | |
| Sa-neoJ | | | *S. albus* CGMCC 4.5716 containing pSET152::P*_neo_*J | | | | This study | | |
| Sa-kasO*J | | | *S. albus* CGMCC 4.5716 containing pSET152::P*_kasO*_*J | | | | This study | | |
| Sa-neoBIII-E | | | *S. albus* CGMCC 4.5716 containing pSET152::P*_neo_*BIII-E | | | | This study | | |
| Sa-kasO*BIII-E | | | *S. albus* CGMCC 4.5716 containing pSET152::P*_kasO*_*BIII-E | | | | This study | | |
| Sa-neoD-F | | | *S. albus* CGMCC 4.5716 containing pSET152::P*_neo_*D-F | | | | This study | | |
| Sa-kasO*D-F | | | *S. albus* CGMCC 4.5716 containing pSET152::P*_kasO*_*D-F | | | | This study | | |
| Sa-neoH-I | | | *S. albus* CGMCC 4.5716 containing pSET152::P*_neo_*H-I | | | | This study | | |
| Sa-kasO*H-I | | | *S. albus* CGMCC 4.5716 containing pSET152::P*_kasO*_*H-I | | | | This study | | |
| Sa-neoBII-BI | | | *S. albus* CGMCC 4.5716 containing pSET152::P*_neo_*BII-BI | | | | This study | | |
| Sa-kasO*BII-BI | | | *S. albus* CGMCC 4.5716 containing pSET152::P*_kasO*_*BII-BI | | | | This study | | |
| Sa-neoC | | | *S. albus* CGMCC 4.5716 containing pSET152::P*_neo_*C | | | | This study | | |
| Sa-kasO*C | | | *S. albus* CGMCC 4.5716 containing pSET152::P*_kasO*_*C | | | | This study | | |
| Sa-D-C | | | *S. albus* CGMCC 4.5716 containing pSET152::D-C | | | | This study | | |
| Sa-mini-cluster | | | *S. albus* CGMCC 4.5716 containing pSET152::mini-cluster | | | | This study | | |
| Sa-sal | | | *S. albus* CGMCC 4.5716 containing pBAC::sal | | | | This study | | |
| Str-99-mini-cluster | | | The streptomycin-resistant mutant Str-99 containing pSET152::mini-cluster | | | | This study | | |
| Str-99-kasO*J | | | The streptomycin-resistant mutant Str-99 containing pSET152::P*_kasO*_*J | | | | This study | | |
| ***Escherichia coli*** | | |  | | | |  | | |
| JM109 | | | F, *proA^+^B^+^*, *lacI^q^*, *Δ(lacZ)M15/ (lac-proAB)*, *gyrA96*, *recA1*, *relA1*, *endA1*, *hsdR17* | | | | Sambrook and Russell, 2001 | | |
| ET12567 | | | *dam, dcm, hsdS, cat, tet* | | | | MacNeil et al., 1992 | | |
| BW25113 | | | K-12 derivative; ΔaraBAD, ΔrhaBAD | | | | Datsenko and Wanner, 2000 | | |
| ***Bacillus cereus*** | | | |  | | |  | | |
| CGMCC 1.1626 | | | | An indicator strain for bioassays | | | CGMCC | | |
| **Plasmids** | | | |  | | |  | | |
| pUZ8002 | | | | *tra*, *neo*, RP4 | | | Paget et al., 1999 | | |
| pIJ790 | | | | λ-Red (*gam*, *bet*, *exo*), *cat*, *araC*, *rep*101^ts^ | | | Gust et al., 2003 | | |
| pUC119::neo | | | | pUC119 containing kanamycin resistance gene (*neo*) | | | Li et al., 2009 | | |
|  | | | |  | | | |  | |
| pDR4-K* | | | | Derived from pSET152, with the constitutive promoter P_kasO*_ | | | | Wang et al., 2013 | |
| pSET152::P*_kasO*_*metK | | | | pSET152 containing *metK* and P*_kasO*_* | | | | This study | |
| pSET152::P*_hrdB_*J | | | | pSET152 containing *salJ* and P*_hrdB_* | | | | This study | |
| pSET152::P*_neo_*J | | | | pSET152 containing *salJ* and P*_neo_* | | | | This study | |
| pSET152::P*_kasO*_*J | | | | pSET152 containing *salJ* and P*_kasO*_* | | | | This study | |
| pSET152::P*_neo_*BIII-E | | | | pSET152 containing *salBIII-E* and P*_neo_* | | | | This study | |
| pSET152::P*_kasO*_*BIII-E | | | | pSET152 containing *salBIII-E* and P*_kasO*_* | | | | This study | |
| pSET152::P*_neo_*D-F | | | | pSET152 containing *salD-F* and P*_neo_* | | | | This study | |
| pSET152::P*_kasO*_*D-F | | | | pSET152 containing *salD-F* and P*_kasO*_* | | | | This study | |
| pSET152::P*_neo_*H-I | | | | pSET152 containing *salH-I* and P*_neo_* | | | | This study | |
| pSET152::P*_kasO*_*H-I | | | | pSET152 containing *salH-I* and P*_kasO*_* | | | | This study | |
| pSET152::P*_neo_*BII-BI | | | | pSET152 containing *salBII-BI* and P*_neo_* | | | | This study | |
| pSET152::P*_kasO*_*BII-BI | | | | pSET152 containing *salBII-BI* and P*_kasO*_* | | | | This study | |
| pSET152::P*_neo_*C | | | | pSET152 containing *salC* and P*_neo_* | | | | This study | |
| pSET152::P*_kasO*_*C | | | | pSET152 containing *salC* and P*_kasO*_* | | | | This study | |
| pUC18::D-C | | | | pUC18 containing *salD-C* cassette | | | | This study | |
| pSET152::D-C | | | | pSET152 containing *salD-C* cassette | | | | This study | |
| pSET152::mini-cluster | | | | pSET152::D-C containing *salJ-ccr* cassette | | | | This study | |
|  | | | |  | | | |  | |
|  | | | |  | | |  | | |
|  | | | |  | | |  | | |
| pBAC::sal | Derived from pBeloBac11 containing *sal* gene cluster, *oriT*, *attP*(ΦC31)-*int*(ΦC31) cassette and replacing the chloramphenicol resistance gene (*cat*) with apramycin resistance gene (*aac(3)IV*) | | | | | | | Our lab | |

**Table S2** Primers used in this study

| **Primers** | **Sequence (5′→3′)** | | **Purpose** | |
| --- | --- | --- | --- | --- |
| P*_kasO_*_*_-F | GGAATTCTGTTCACATTCGAACGGTCTCTG | | Amplification of P*_kasO_*_*_ | |
| P*_kasO*_*-R | AACTCCCCCAGTCCTGCACGCT | | Amplification of P*_kasO_*_*_ | |
| P*_hrdB_*-F | GGAATTCCCGCCTTCCGCCGGAACG | | Amplification of P*_hrdB_* | |
| P*_hrdB_*-R | GAACAACCTCTCGGAACGTTGA | | Amplification of P*_hrdB_* | |
| P*_neo_*-F | GGAATTCGGATCCCCTGGATACC | | Amplification of P*_neo_* | |
| P*_neo_*-R | GCGAAACGATCCTCATCCTGT | | Amplification of P*_neo_* | |
| metK-F | GTGTCCCGCCGTCTGTTCACC | | Amplification of *metK* | |
| metK-R | GCTCTAGACGTGGAGCCGCTCAGAGCCCC | | Amplification of *metK* | |
| salJ-F | GTGACTCTTTACGACCGTGACA | | Amplification of *salJ* | |
| salJ-R | GCTCTAGAGGCTCCACACCCCTGAGTAAC | | Amplification of *salJ* | |
| salBIIIGIE-F | ATGCAGGACGAGCAGAAGC | | Amplification of *salBIII-E* genes | |
| salBIIIGIE-R | GCTCTAGAGCCGCGATTCACGCCAGAG | | Amplification of *salBIII-E* genes | |
| salDF-F | ATGCCTGCCTCAAAGTCTTCCTG | | Amplification of *salD-F* genes | |
| salDF-R | GCTCTAGAGGTTCCGGTGGGCGTTTCGTC | | Amplification of *salD-F* genes | |
| salHI-F | TTGGCGCCCAACGAGTACGCAA | | Amplification of *salH-I* genes | |
| salHI-R | GCTCTAGAGGGCCGTACCGCTGCGTTCT | | Amplification of *salH-I* genes | |
| salBIIBI-F | ATGCTGGATGAGGCGGCACT | | Amplification of *salBII-BI* genes | |
| salBIIBI-R | ATCGATCGATCCAGTGTTCTTCCGGGGGTAA | | Amplification of *salBII-BI* genes | |
| salC-F | GTGCCCGCGGCCGACGGA | | Amplification of *salC* | |
| salC-R | GCTCTAGAGCACATTCCCGGCTGACTTCTT | | Amplification of *salC* | |
| MC-J-F | CTGGACATGATAAACATCTCGGCTCTCTGATGTTCACATTCGAACGGTCTCTG | | Amplification of P*_kasO*_*::*salJ* | |
| MC-J-R | TCCTGCGTTATCCCGGGTTATCGGACCTCAGGCTCCACACCCCTGAGTAAC | | Amplification of P*_kasO*_*::*salJ* | |
| MC-DF-F | ACCATTGCGAACGATGAAAGCATTGGCAACTGTTCACATTCGAACGGTCTCTG | | Amplification of P*_kasO*_*::*salD-F* | |
| MC-DF-R | | GCAGCGGTACGGCCCGTTCCGGTGGGCGTTTCGTC | | Amplification of P*_kasO*_*::*salD-F* |
| MC-HI-F | | AACGCCCACCGGAACGGGCCGTACCGCTGCGTTCT | | Amplification of P*_neo_*::*salH-I* |
| MC-HI-R | | CATCGCTTCACGATCTTCGCTTTCGTTAATATGGCAGATCCCCTGGATACCGCTCGC | | Amplification of P*_neo_*::*salH-I* |
| MC-BIIIGIE-F | | TGCCATATTAACGAAAGCGAAGATCGTGAAGCGATGTGTTCACATTCGAACGGTCTCTG | | Amplification of P*_kasO*_*::*salBIII-E* |
| MC-BIIIGIE-R | | TCAGCCGGGAATGTGCGCCGCGATTCACGCCAGAGA | | Amplification of P*_kasO*_*::*salBIII-E* |
| MC-C-F | | GCGTGAATCGCGGCGCACATTCCCGGCTGACTTCTT | | Amplification of P*_neo_*::*salC* |
| MC-C-R | | TCCCCGAAAAGTGCCACCTGACGTCTAAGAGATCCCCTGGATACCGCTCGC | | Amplification of P*_neo_*::*salC* |
| ccr-Pneo-F | | TCAGAGAGCCGAGATGTTTATCATGTCCAGGATCCCCTGGATACCGCTCGC | | Amplification of P*_neo_* for *ccr* |
| ccr-Pneo-R | | CAGGATGTCCTTCACGCGAAACGATCCTCATCCTGTC | | Amplification of P*_neo_* for *ccr* |
|  | |  |  | |
| MC-ccr-F | | TGAGGATCGTTTCGCGTGAAGGACATCCTGGACGCG | Amplification of *ccr* | |
| MC-ccr-R | | GTTGCCAATGCTTTCATCGTTCGCAATGGTTCAGACGTTCCGGAAGCGGTTG | Amplification of *ccr* | |
| pUC18-F | | GGTATCCAGGGGATCTCTTAGACGTCAGGTGGCACTT | Amplification of pUC18 vector | |
| pUC18-R | | TGAGGTCCGATAACCCGGGATAACGCAGGAAAGAACATG | Amplification of pUC18 vector | |
| pUC18-R2 | | GTTGCCAATGCTTTCATCGTTCGCAATGGTGATATCTGAGGTCCGATAACCCGGGATA | Amplification of pUC18 vector | |
| Targeting 152-F | | TCTTAGACGTCAGGTGGCACTTTTCGGGGAAATGTGCGCCACACAACATACGAGCCGGA | Amplification of pSET152 vector | |
| Targeting 152-R | | GGGATAACGCAGGAAAGAACATGTGAGCAAAAGGCCAGCGTCGACCTGCAGCCCAAGC | Amplification of pSET152 vector | |
| MCS-F | | GGATGTGCTGCAAGGCGATT | Verification for pSET152::mini-cluster | |
|  | |  |  | |
| salJ-in-R | | GGGTGGCTCAGGCGGCAGTCG | Verification for pSET152::mini-cluster | |
| salE-in-F | | CGATGAACAAGCACTGGACGG | Verification for pSET152::mini-cluster | |
| MCS-R | | CTCATTAGGCACCCCAGGCT | Verification for pSET152::mini-cluster | |
| qhrdB-F | | TCAAGCGCGAACTGGAGATCAT | *hrdB* RT-qPCR | |
| qhrdB-R | | GCGGATCAGACCGAGGTTGC | *hrdB* RT-qPCR | |
| qmetK-F | | GATGCCGCTCCCGATCAGCC | *metK* RT-qPCR | |
| qmetK-R | | GGTCGATGTCCGAGGCGTGC | *metK* RT-qPCR | |
| qJ-F | | CCGCCTACGGCTACACCAACC | *salJ* RT-qPCR | |
| qJ-R | | TCGAAGTGCAGGTCCATCGGC | *salJ* RT-qPCR | |
| qAI-F | | ACCGCATCGAGATCCTTCAGC | *salAI* RT-qPCR | |
| qAI-R | | CCGCATCCTCAAGCGACAACG | *salAI* RT-qPCR | |
| qBIII-F | | CAAGGAGATCGTCGCGGAATAC | *salBIII* RT-qPCR | |
| qBIII-R | | ACCTCGGCGGTGACATTGCTG | *salBIII* RT-qPCR | |
| qD-F | | TCGCCGAGGGTGAGTCCGTGT | *salD* RT-qPCR | |
| qD-R | | CCGAAGCCCATGTGGTGGTTGT | *salD* RT-qPCR | |
| qH-F | | TCTGCGACCGCGTCAGCATC | *salH* RT-qPCR | |
| qH-R | | GTTGTCCACGTCGAGTTTGAGG | *salH* RT-qPCR | |
| qBII-F | | CGAGGGCCTGCTCAAGCTGT | *salBII* RT-qPCR | |
| qBII-R | | CGATCTCCACGGCGTTGCTG | *salBII* RT-qPCR | |
| qC-F | | CGGTCTACGGACACGGCATGA | *salC* RT-qPCR | |
| qC-R | | GCCCGGGTAGCAGATGTCCT | *salC* RT-qPCR | |
| qccr-F | | CCGACGGGCACAACGACACCA | *ccr* RT-qPCR | |
| qccr-R | | TGGTCGGGCTTGGGCATCAG | *ccr* RT-qPCR | |


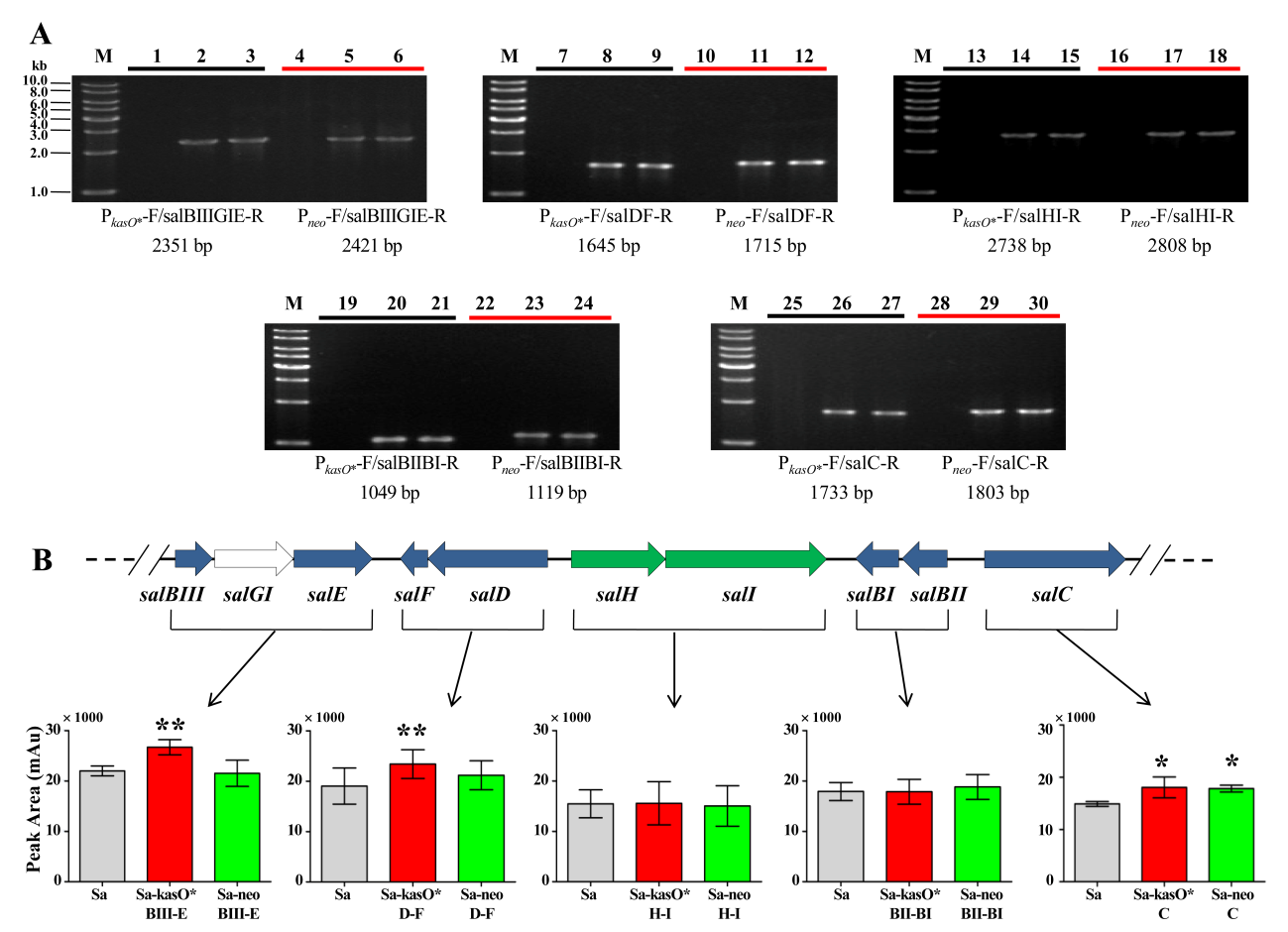
**Fig. S1.** PCR analysis of the gene modules in the overexpression strains (A) and the HPLC analysis of salinomycin production (B). (A) M, DNA ladder; Lanes 1, 4, 7, 10, 13, 16, 19, 22, 25 and 28: negative controls; Lanes 2-3, 5-6, 8-9, 11-12, 14-15, 17-18, 20-21, 23-24, 26-27 and 29-30: PCR products using genomic DNAs of strains Sa-kasO*BIII-E, Sa-neoBIII-E, Sa-kasO*D-F, Sa-neoD-F, Sa-kasO*H-I, Sa-neoH-I, Sa-kasO*BII-BI, Sa-neoBII-BI, Sa-kasO*C and Sa-neoC as template, respectively.

**
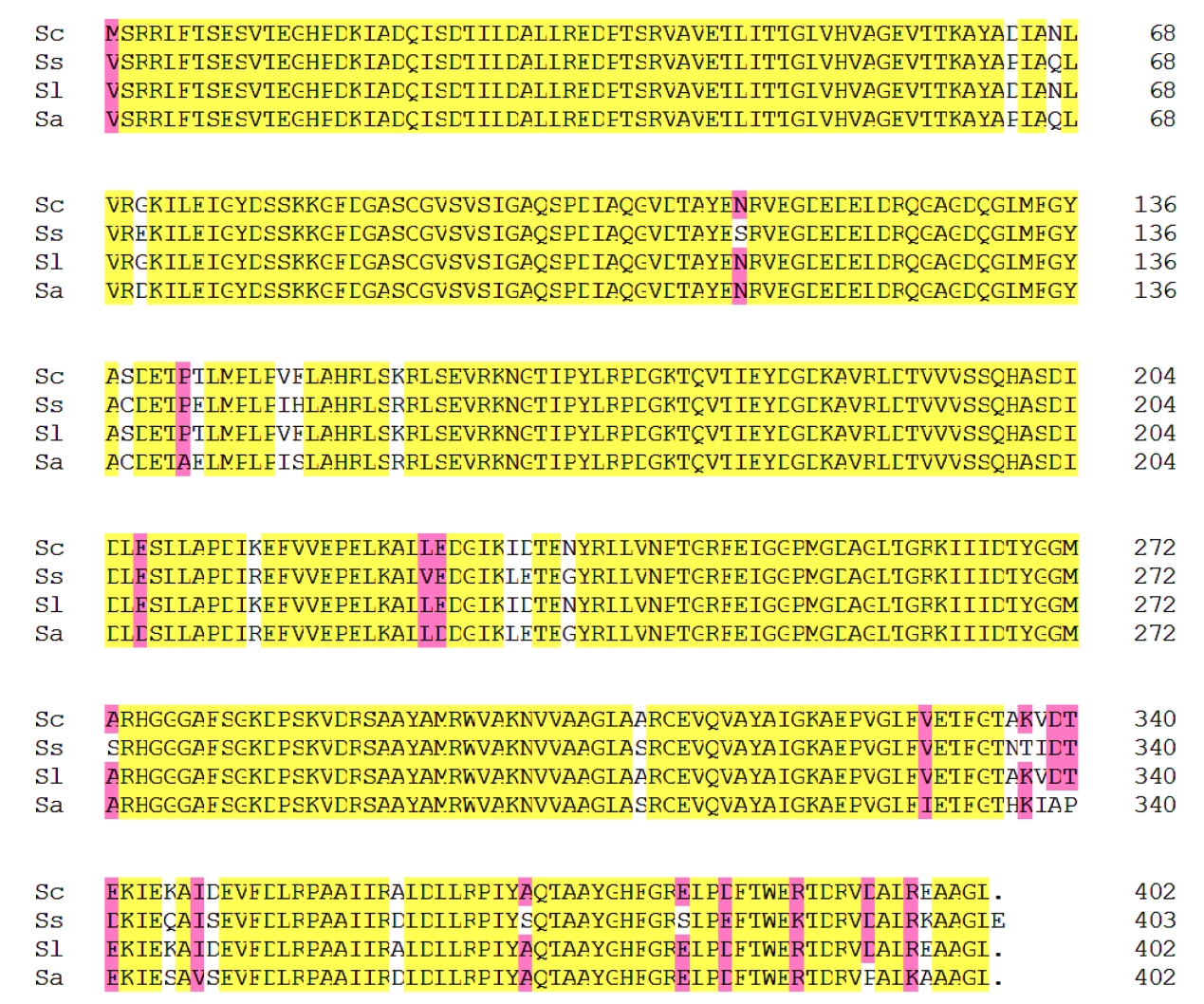
**

**Fig. S2.** Sequence alignment of MetK from different *Streptomyces* (Sc, *Streptomyces coelicolor*. Ss, *Streptomyces* *spectabilis*. Sl, *Streptomyces* *lividans*. Sa, *Streptomyces albus*).
